# Single-cell analysis reveals the pan-cancer invasiveness-associated transition of adipose-derived stromal cells into COL11A1-expressing cancer-associated fibroblasts

**DOI:** 10.1371/journal.pcbi.1009228

**Published:** 2021-07-20

**Authors:** Kaiyi Zhu, Lingyi Cai, Chenqian Cui, Juan R. de los Toyos, Dimitris Anastassiou

**Affiliations:** 1 Department of Systems Biology, Columbia University, New York, New York, United States of America; 2 Department of Electrical Engineering, Columbia University, New York, New York, United States of America; 3 Center for Cancer Systems Therapeutics, Columbia University, New York, New York, United States of America; 4 Immunology Department, School of Medicine and Health Sciences, University of Oviedo, Oviedo, Spain; 5 Herbert Irving Comprehensive Cancer Center, Columbia University, New York, New York, United States of America; University of Chicago, UNITED STATES

## Abstract

During the last ten years, many research results have been referring to a particular type of cancer-associated fibroblasts associated with poor prognosis, invasiveness, metastasis and resistance to therapy in multiple cancer types, characterized by a gene expression signature with prominent presence of genes *COL11A1*, *THBS2* and *INHBA*. Identifying the underlying biological mechanisms responsible for their creation may facilitate the discovery of targets for potential pan-cancer therapeutics. Using a novel computational approach for single-cell gene expression data analysis identifying the dominant cell populations in a sequence of samples from patients at various stages, we conclude that these fibroblasts are produced by a pan-cancer cellular transition originating from a particular type of adipose-derived stromal cells naturally present in the stromal vascular fraction of normal adipose tissue, having a characteristic gene expression signature. Focusing on a rich pancreatic cancer dataset, we provide a detailed description of the continuous modification of the gene expression profiles of cells as they transition from *APOD*-expressing adipose-derived stromal cells to *COL11A1*-expressing cancer-associated fibroblasts, identifying the key genes that participate in this transition. These results also provide an explanation to the well-known fact that the adipose microenvironment contributes to cancer progression.

## Author summary

Computational analysis of rich gene expression data at the single-cell level from cancer biopsies can lead to biological discoveries about the nature of the disease. Using a computational methodology that identifies the gene expression profile of the dominant cell population for a particular cell type in the microenvironment of tumors, we observed that there is a remarkably continuous modification of this profile among patients, corresponding to a cellular transition. Specifically, we found that the starting point of this transition has a unique characteristic signature corresponding to cells that are naturally residing in normal adipose tissue. We also found that the endpoint of the transition has another characteristic signature corresponding to a particular type of cancer-associated fibroblasts with prominent expression of gene *COL11A1*, which has been found strongly associated with invasiveness, metastasis and resistance to therapy in multiple cancer types. Our results provide an explanation to the well-known fact that the adipose tissue contributes to cancer progression, shedding light on the biological mechanism by which tumor cells interact with the adipose microenvironment. We provide a detailed description of the changing profile during the transition, identifying associated genes as potential targets for pan-cancer therapeutics inhibiting the underlying mechanism.

## Introduction

This work investigates, using computational analysis of rich single-cell datasets from many patients, the nature and origin of a particular type of cancer-associated fibroblasts (CAFs) that has been found to be strongly associated with invasiveness, metastasis, resistance to therapy, and poor prognosis, in multiple cancer types. These fibroblasts can be identified by their characteristic signature with prominent presence of collagen *COL11A1* and several other co-expressed genes such as *THBS2* and *INHBA*. There are indications that the generation of those CAFs is part of a universal biological process in cancer that plays essential roles in cancer progression. Therefore, the driving vision for this research has been that it may provide testable hypotheses for the development of pan-cancer therapeutics targeting the biological mechanisms responsible for the creation of those CAFs. As described below, to achieve this task we used both established techniques for studying the dynamic changes in gene expression of cells associated with lineages, such as trajectory inference, as well as complementary computational approaches with novel application in single-cell data analysis. These techniques allowed the precise identification of the expression profile of the origin of the underlying cellular transition as a particular cell type of adipose derived stromal/stem cells (ASCs). We also independently validated the presence of those ASCs as naturally occurring, by applying the same computational methods in other available datasets of normal adipose tissue. In the remaining part of this section we provide introductory information about the *COL11A1*-expressing CAFs, explain the motivation for our choice of computational methods, and provide evidence for their advantages and unique capabilities analyzing the particular data sets that we used.

These CAFs were first identified in 2010 [1] by their cancer stage-associated signature. Specifically, a *COL11A1*/*INHBA*/*THBS2*-expressing gene signature was found to be present only after a particular staging threshold, different in each cancer type, was reached. For example, it only appeared in ovarian cancer of at least stage III; in colon cancer of at least stage II; and in breast cancer of at least invasive stage I (but not in carcinoma in situ). We had observed the striking consistency of that signature across cancer types, which was obvious at that time from bulk microarray data. For example, Table 1 shows the top 15 genes ranked in terms of fold change for three different cancer types (breast [2], ovarian [3], pancreatic [4]) using data provided in papers published independently. The breast cancer data compare invasive ductal carcinoma with ductal carcinoma in situ (supplementary data 3, “up in IDC” of the paper [2]); the ovarian cancer data compare metastatic tissue in the omentum with primary tumor (Table 2 of the paper [3]); and the pancreatic data compare whole tumor tissue with normal pancreatic tissue (Table 1 of the paper [4]). The four genes *COL11A1*, *INHBA*, *THBS2*, *COL5A2* appear among the top 15 in all three sets (*P* = 6×10^−23^ by multi-set intersection test [5]). The actual *P* value is much lower than that, because, in addition to the above overlap, ten additional genes (*COL10A1*, *COL1A1*, *COL5A1*, *FAP*, *FBN1*, *FN1*, *LOX*, *MFAP5*, *POSTN*, *SULF1*) appear among the top 15 in at least two of the three sets (and are highly ranked in all three sets anyway). This similarity demonstrates that the signature is well-defined and associated with a universal biological mechanism in cancer.

**Table 1:** Top 15 ranked genes in terms of fold change (FC) for three different cancer types revealing the signature of the *COL11A1*-expressing cancer-associated fibroblasts. Shown are the rankings, reported by the authors, for breast, ovarian and pancreatic cancers. We eliminated multiple entries of the same gene (keeping the one that appears first) and dashes. Genes shared in all three cancer types are highlighted in green, while genes appearing twice are highlighted in yellow.

	Breast		Ovarian		Pancreatic	
Rank	Gene	FC	Gene	FC	Gene	Log2FC
1	COL11A1	6.5	COL11A1	8.23	INHBA	5.15
2	COL10A1	4.07	COL1A1	5.67	COL10A1	5
3	MFAP5	3.73	TIMP3	5.52	POSTN	4.92
4	LRRC15	3.61	FN1	5.4	SULF1	4.63
5	INHBA	3.44	INHBA	4.94	COL8A1	4.6
6	FBN1	3.43	EFEMP1	4.86	COL11A1	4.4
7	SULF1	3.35	DSPG3	4.36	CTHRC1	4.38
8	GREM1	3.35	COL5A2	4.07	COL1A1	4.12
9	COL5A2	3.22	LOX	4.03	THBS2	3.97
10	LOX	3.22	MFAP5	4.01	HNT	3.9
11	COL5A1	3.08	POSTN	3.97	CSPG2	3.87
12	THBS2	2.99	COL5A1	3.95	WISP1	3.8
13	LAMB1	2.97	THBS2	3.91	FN1	3.69
14	FAP	2.96	FBN1	3.9	COMP	3.53
15	SPOCK	2.91	FAP	3.84	COL5A2	3.38

We had also found that gene *COL11A1* serves as a proxy of the full signature, in the sense that it is the only gene from which all other genes of the signature are consistently top-ranked in terms of the correlation of their expression with that of *COL11A1*. Accordingly, we had identified a *COL11A1*-correlated pan-cancer gene signature, listed in table 4 of [1], which we deposited in the Molecular Signatures Database (MSigDB). We had referred to those CAFs as MAFs (“metastasis-associated fibroblasts”), because their presence suggests that metastasis is imminent. To avoid any inaccurate interpretation of the term as implying that such fibroblasts are markers of metastasis that has occurred already, here we refer to them as “*COL11A1*-expressing CAFs.”

Since then, many research results were published connecting one of the genes *COL11A1*, *INHBA*, *THBS2* with poor prognosis, invasiveness, metastasis, or resistance to therapy, in various cancer types [6–15].

Furthermore, several designated tumor subtypes were identified in individual cancer types as a result of the presence of those pan-cancer CAFs. For example, the top 15 genes distinguishing the ovarian "mesenchymal subtype" according to [16] are *POSTN*, *COL11A1*, *THBS2*, *COL5A2*, *ASPN*, *FAP*, *MMP13*, *VCAN*, *LUM*, *COL10A1*, *CTSK*, *COMP*, *CXCL14*, *FABP4*, *INHBA*. Similarly, the 24 characterizing genes of the "activated stroma subtype" of pancreatic cancer in Fig 2 of [17] are *SPARC*, *COL1A2*, *COL3A1*, *POSTN*, *COL5A2*, *COL1A1*, *THBS2*, *FN1*, *COL10A1*, *COL5A1*, *SFRP2*, *CDH11*, *CTHRC1*, *PNDC1*, *SULF1*, *FAP*, *LUM*, *COL11A1*, *ITGA11*, *MMP11*, *INHBA*, *VCAN*, *GREM1*, *COMP*. In both of these examples, these gene lists are clearly due to the presence of the *COL11A1*/*INHBA*/*THBS2*-expressing CAFs and therefore these are not cancer-type specific subtype signatures.

To computationally investigate the origin of those CAFs, we reasoned that analysis of rich datasets from single-cell RNA sequencing (scRNA-seq) provides unique opportunities for tracking the trajectories of cell differentiation lineages. There are several single-cell trajectory inference methods [18] performing “trajectory inference analysis,” ordering cells along a trajectory based on similarities in expression patterns.

In particular, we identified one exceptionally rich dataset [19] from pancreatic ductal adenocarcinoma, containing gene expression profiles from 24 tumor samples and 11 normal control samples. We found that several among the 24 tumor samples contained populations of cells strongly co-expressing *COL11A1*, *THBS2* and *INHBA*, while none of the normal samples contained such cells. We also observed that the prominence of this co-expression signature varied significantly among the tumor samples, having only hints of their presence in some of them, suggesting that the corresponding patients were at various stages of the generation of *COL11A1*-expressing CAFs. This provides an opportunity to perform additional complementary computational analysis by comparing the prevalent fibroblastic cell populations across the tumor samples, and comparing them with those in the normal samples.

Therefore, in this paper we also used attractor analysis (Materials and Methods) in a novel manner for the analysis of rich scRNA-seq data. The unsupervised attractor algorithm [20] iteratively finds co-expression signatures converging to “attractor metagenes” pointing to the core (“heart”) of co-expression. Each attractor metagene is defined by a ranked set of genes along with scores determining their corresponding strengths within the signature, so the top-ranked genes are the most representative of the signature. The attractor algorithm has previously been used successfully for identifying features useful for breast cancer prognosis [21,22].When applied on single cell data from a sample, it identifies the gene expression profiles of the dominant cell populations in the sample, and the algorithm is designed to ensure that all the top-ranked genes are co-expressed in the same cells. The purpose of the attractor algorithm is not to classify cells into mutually exclusive subsets. Instead, it identifies the genes at the core of co-expression signatures representing cellular populations from single-cell data, and it provides information that cannot be deduced with traditional clustering methods (see Discussion).

When we applied the attractor algorithm separately in each of the normal samples, we identified a set of nearly identical attractor signatures, corresponding to a type of adipose-derived stromal/stem cells (ASCs) naturally present in the stromal vascular fraction (SVF) of normal adipose tissue, expressing a unique characteristic signature containing fibroblastic markers such as *LUM* and *DCN* as well as adipose-related genes, such as *APOD*, *CFD* and *MGP*.

When we applied the algorithm in each of the tumor samples, we found a set of signatures that were changing in a remarkably continuous manner across the samples, some of them being very similar to those of the normal samples, while others are similar to the *COL11A1*-based signature. This suggests that the signatures undergo a gradual change as the transition proceeds, starting from the state of the normal ASCs and passing through a continuum of intermediate states. These results were consistent with those found by applying trajectory inference analysis, but they provided additional significant information based on their unique capabilities. Accordingly, this method demonstrated that there is a continuous “ASC to *COL11A1*-expressing CAF transition.”

This finding explains the stage association of the *COL11A1*-expressing signature as resulting from the interaction of tumor cells with the adipose microenvironment: Indeed, adipose tissue is encountered when ovarian cancer cells reach the omentum (stage III); after colon cancer has grown outside the colon (stage II); and in breast cancer from the beginning of the spread (stage I, but not in situ stage 0).

Finally, we validated our results in other cancer types (head and neck, ovarian, lung, breast), suggesting the pan-cancer nature of the ASC to *COL11A1*-expressing CAF transition.

## Results

### ASC to *COL11A1*-expressing CAF transition identified in pancreatic ductal adenocarcinoma (PDAC)

The PDAC dataset [19] consists of 57,530 scRNA-seq profiles from 24 PDAC tumor samples (T1-T24) and 11 normal samples (N1-N11). To find the expression profile of the dominant fibroblastic population in each sample, we applied the attractor algorithm on the set of identified mesenchymal cells (Materials and Methods). All samples (11 normal and 23 tumor samples, excluding sample T20 as it did not contain identified fibroblasts) yielded strong co-expression signatures involving many genes with big overlap among them. Genes *LUM*, *DCN*, *FBLN1*, *MMP2*, *SFRP2* and *COL1A2* appear in the top 100 genes in at least 33 out of the 34 samples (S1 Table), revealing a strong similarity shared by all those fibroblastic expression profiles. This strong overlap is consistent with the continuous transition process, as described below.

#### Dominant fibroblastic population in the normal pancreatic samples is adipose-derived

There is a striking similarity among the attractor profiles (Materials and Methods) of the eleven normal pancreatic samples, indicating that they represent a stable and normally occurring cell population. Specifically, there are 12 genes commonly shared among the top 30 genes in the attractors of at least ten of the eleven normal samples (Table 2), of which four genes are shared among all the samples (*P* = 3×10^−113^ by multi-set intersection test [5]). In addition to fibroblastic markers, there are several strongly expressed adipose-related or stemness-related genes in the list, such as *APOD*, *CXCL12*, and *DPT*, revealing that they are ASCs. Consistently, Gene Set Enrichment Analysis (GSEA) of these 12 commonly shared genes identified the most significant enrichment (FDR *q* value = 2.16 ×10^−19^) in the “BOQUEST_STEM_CELL_UP” dataset of genes upregulated in stromal stem cells from adipose tissue versus the non-stem counterparts [23].

**Table 2:** Top 30 genes of the identified attractors for each pancreatic normal sample (N1-N11). 12 commonly shared genes in at least ten of the eleven normal samples are highlighted.

Rank	N1	N2	N3	N4	N5	N6	N7	N8	N9	N10	N11
**1**	DCN	LUM	LUM	C7	APOD	LUM	PTGDS	C7	DCN	MMP2	LUM
**2**	LUM	DCN	FBLN1	FBLN5	DPT	DCN	APOD	LUM	LUM	APOD	DCN
**3**	C7	C7	C7	LUM	FBLN5	FBLN1	LUM	DCN	C7	LUM	FBLN1
**4**	FBLN1	FBLN1	PTGDS	DCN	PDGFRA	ADH1B	FBLN1	APOD	FBLN1	EFEMP1	SFRP2
**5**	MGP	APOD	C1S	APOD	CXCL12	DPT	C7	FBLN1	APOD	CTSK	CFD
**6**	C1S	MGP	DPT	PTGDS	LUM	ABCA8	ADH1B	SFRP2	SFRP2	SFRP2	APOD
**7**	CCDC80	C1S	PDGFRA	FBLN1	COL6A3	C3	DPT	PTGDS	SERPINF1	PLTP	MGP
**8**	PTGDS	DPT	APOD	C1R	PTGDS	APOD	COL6A3	CCDC80	PTGDS	MGST1	SERPINF1
**9**	DPT	CCDC80	SFRP2	DPT	C7	MMP2	EFEMP1	FBLN5	GSN	LSP1	CCDC80
**10**	C1R	PTGDS	DCN	SRPX	CCDC80	C1S	PDGFRA	C1S	C1S	FBLN1	C3
**11**	APOD	FBLN5	CXCL12	FMO2	CFD	C7	CXCL12	CXCL12	SEPP1	SPON2	ADH1B
**12**	SEPP1	SEPP1	C1R	SEPP1	MRC2	PTGDS	SCN7A	C3	CCDC80	PTGDS	PTGDS
**13**	FBLN5	COL1A2	COL6A3	CXCL12	FGF7	SFRP2	MMP2	CFD	DPT	SVEP1	C7
**14**	CXCL12	SFRP2	ADH1B	CYR61	SFRP2	FBLN5	MEG3	C1R	OLFML3	CXCL12	C1S
**15**	EFEMP1	SRPX	SPON2	SFRP2	MARCKS	C1R	C1S	MGP	FBLN5	SCN7A	CST3
**16**	COL1A2	SERPINF1	CFD	CLEC11A	LRP1	CXCL12	OLFML3	CFH	C1R	COL6A3	C1R
**17**	SFRP2	OLFML3	LAMA2	PDGFRA	FMO2	CST3	SVEP1	COL6A3	PTN	CCDC80	CXCL14
**18**	ALDH1A1	CST3	C3	NR2F1	NR2F1	MGP	DCN	SRPX	MGP	COLEC11	MMP2
**19**	CFD	MEG3	FBLN5	C1S	TNXB	CCDC80	SFRP2	EFEMP1	ALDH1A1	PDGFRA	GPNMB
**20**	COL6A3	C1R	ABCA8	ABCA8	DCN	MRC2	MRC2	SEPP1	PDGFRA	HBP1	S100A4
**21**	EMP1	MFAP4	LRP1	CCDC80	LOX	COL1A2	FBLN5	PDGFRA	COL6A2	CYGB	DPT
**22**	PCOLCE	RARRES2	SLIT2	PTN	C1R	CFD	C3	DPT	CST3	ARSK	MFAP4
**23**	C3	PCOLCE	CFH	SERPINF1	IGFBP3	SPRY1	COL1A2	CXCL14	COL6A3	SH3GL1	COL6A2
**24**	SRPX	CFH	SRPX	SVEP1	HEG1	SMOC2	ABCA8	ADH1B	CXCL14	OAF	FBLN5
**25**	SERPINF1	CXCL12	COL1A2	CFD	RP11-572C15.6	GSN	SRPX	NEGR1	C3	BMP1	SMOC2
**26**	ANXA1	FGF7	BOC	LAMB1	F3	COL6A2	ACVRL1	COL6A2	CXCL12	LAMA2	ABCA8
**27**	CYR61	PDGFRA	FSTL1	FTL	ADAMTSL3	CFH	TIMP2	BOC	MMP2	GPC3	FMO2
**28**	CST3	COL6A3	SVEP1	ANTXR2	STK17B	OLFML3	LAMA2	OLFML3	PCOLCE	TMEM67	RP11-572C15.6
**29**	RARRES2	ALDH1A1	ABCA9	COL6A3	EMP1	PDGFRA	DAB2	EMP1	IGF1	C1R	PCOLCE
**30**	PDGFRA	SPRY1	CYR61	MGP	MPZL1	PCOLCE	NR2F1	LAMA2	ABCA8	PLXDC1	SEPP1

To investigate the nature of this ASC population, we referred to recent results from single-cell analysis of general human adipose tissue [24]. We applied the attractor algorithm on the dataset with the single-cell expression profiles of all 26,350 cells taken from the SVF of normal adipose tissue from 25 samples, and compared the identified attractor with the “consensus attractor” (Materials and Methods) of the 11 normal pancreatic samples, which represented the main state of the normal fibroblastic population (Table 3). There are 14 overlapping genes between the top 30 gene lists (*P* = 10^−33^ by hypergeometric test), and most of the non-highlighted genes in each column are still ranked highly in the other column. This extreme similarity of the two gene expression profiles indicates that they correspond to the same naturally occurring cell population. Furthermore, excluding the general fibroblastic markers *LUM* and *DCN*, we found that gene *APOD* (Apolipoprotein D) has the highest average ranking in Table 3, and is top-ranked in the independently found SVF fibroblastic population of cluster VP4 (supplementary file 20) of [24]. Therefore, we selected *APOD* as the representative marker for the ASC population.

**Table 3:** Comparison of the attractors (top 30 genes) identified in the SVF of normal adipose tissue (Dataset 1) and in the normal pancreatic samples (Dataset 2). Common genes are highlighted in yellow.

Rank	Dataset 1	Dataset 2	Rank (cont’d)	Dataset 1	Dataset 2
**1**	DCN	LUM	**16**	FOS	PDGFRA
**2**	LUM	DCN	**17**	MGST1	SRPX
**3**	APOD	FBLN1	**18**	COL1A2	COL6A3
**4**	CFD	C7	**19**	COL6A3	ADH1B
**5**	CXCL14	APOD	**20**	LAPTM4A	CFD
**6**	MGP	PTGDS	**21**	CXCL12	OLFLM3
**7**	SERPINF1	SFRP2	**22**	WISP2	SERPINF1
**8**	GSN	C1S	**23**	SRPX	MMP2
**9**	GPX3	CCDC80	**24**	JUN	CST3
**10**	MFAP4	MGP	**25**	MMP2	SEPP1
**11**	PLAC9	DPT	**26**	COL6A2	ABCA8
**12**	S100A13	CXCL12	**27**	C1S	COL1A2
**13**	IGFBP6	C1R	**28**	CCDC80	LAMB1
**14**	DPT	FBLN5	**29**	EGR1	SVEP1
**15**	MFAP5	C3	**30**	PCOLCE	MEG3

#### Establishing the presence of *COL11A1*-expressing CAFs in PDAC tumor samples

Because *COL11A1* serves as proxy of the full signature [1], a reliable test for determining if a sample contains the *COL11A1*-expressing CAFs is to rank all genes in terms of their association, measured by mutual information (Materials and Methods), with *COL11A1* and see if *INHBA* and *THBS2* are top ranked. Indeed, this happens in several tumor samples, as shown in Table 4 for some of them (T23, T11, T6, T15, T18). For each sample, the shown genes are co-expressed in the same cells, because of the high correlations in a single-cell dataset.

**Table 4:** Ranked *COL11A1*-associated genes in five PDAC samples. MI = Mutual Information.

Rank	T23	MI	T11	MI	T6	MI	T15	MI	T18	MI
**1**	COL11A1	1	COL11A1	1	COL11A1	1	COL11A1	1	COL11A1	1
**2**	COL10A1	0.3603	CTHRC1	0.2434	MFAP5	0.2353	MFAP5	0.3198	MFAP5	0.3408
**3**	COL12A1	0.3383	MFAP5	0.2357	FNDC1	0.1997	GJB2	0.2583	SUGCT	0.3379
**4**	COL1A1	0.3187	COL12A1	0.2345	NTM	0.1912	COL10A1	0.2580	COL10A1	0.2899
**5**	THBS2	0.3167	COL10A1	0.2238	COL8A1	0.1877	INHBA	0.2561	C5orf46	0.2753
**6**	COL1A2	0.3099	C1QTNF3	0.2155	TWIST1	0.1714	C1QTNF3	0.2514	PPAPDC1A	0.2668
**7**	COL5A2	0.3003	THBS2	0.2123	COL10A1	0.1619	MATN3	0.2505	NTM	0.2649
**8**	CTHRC1	0.2854	COL1A2	0.2045	THBS2	0.1559	FNDC1	0.2503	COL8A1	0.2534
**9**	FN1	0.2781	COL8A1	0.2018	ITGA11	0.1556	COL8A2	0.2411	INHBA	0.2430
**10**	COL3A1	0.2770	AEBP1	0.2000	PPAPDC1A	0.1305	COL1A1	0.2399	FNDC1	0.2264
**11**	INHBA	0.2746	LUM	0.1989	DIO2	0.1298	COL12A1	0.2351	COL12A1	0.2194
**12**	AEBP1	0.2688	COL1A1	0.1985	IGFL2	0.1178	COL8A1	0.2325	IGFL2	0.2153
**13**	COL5A1	0.2626	FNDC1	0.1963	SUGCT	0.1170	THBS2	0.2292	THBS2	0.2094
**14**	VCAN	0.2457	SFRP2	0.1955	ADAM12	0.1165	NTM	0.2257	CTHRC1	0.2026
**15**	MFAP5	0.2449	GJB2	0.1879	C1QTNF3	0.1165	COL1A2	0.2220	SULF1	0.2015
**16**	MMP11	0.2360	MATN3	0.1817	ITGBL1	0.1109	GREM1	0.2156	COMP	0.1926
**17**	COL8A1	0.2357	COL3A1	0.1740	GREM1	0.1018	FN1	0.2146	STMN2	0.1926
**18**	COL6A3	0.2339	INHBA	0.1696	P4HA3	0.1008	IGFL2	0.2141	WNT2	0.1925
**19**	POSTN	0.2316	DCN	0.1692	INHBA	0.1002	CXCL14	0.2112	MMP11	0.1919
**20**	MFAP2	0.2275	CTGF	0.1691	COL5A1	0.0983	ITGBL1	0.2048	SPOCK1	0.1878

#### Dominant fibroblastic populations in the tumor PDAC samples exhibits a continuous transition from ASCs to *COL11A1*-expressing CAFs

Based on the selection of *APOD* as a representative marker for the ASC population as described previously, we rearranged the attractors of the PDAC tumor samples in terms of descending order of the rank of *APOD* (Table 5) from left to right. There is a remarkable continuity in the shown expression profiles. The samples at the right side of the table include *COL11A1* at increasingly high ranks. The intermediate tumor samples shown in the middle have cells expressing genes that are top-ranked in both the lists on the left as well as on the right. In other words, these cells are in a genuine intermediate state, rather than being a mixture of distinct subtypes (see detailed discussion in Materials and Methods).

**Table 5:** Rearranged PDAC tumor samples showing the continuously changing pattern of the signature profile. Columns are sorted based on *APOD* rankings. Genes *APOD* and *COL11A1* are highlighted in green and red, respectively.

Rank	T2	T13	T14	T19	T3	T10	T15	T18	T7	T6	T12	T4	T24	T1	T5	T22	T11	T21	T23	T9	T16	T17	T8
**1**	LUM	LUM	DCN	SFRP2	MMP2	PDGFRA	SFRP2	DCN	CYP1B1	COL10A1	MMP2	COL10A1	DCN	SFRP2	PDGFRA	COL1A2	LUM	COL10A1	COL1A1	LUM	LUM	COL10A1	COL11A1
**2**	APOD	APOD	APOD	APOD	LUM	HTRA3	LUM	SFRP2	SFRP2	PDGFRA	LUM	SFRP2	LUM	VCAN	CYP1B1	PDGFRA	DCN	CTHRC1	COL1A2	DCN	DCN	CTHRC1	COL10A1
**3**	VCAN	DCN	LUM	LUM	APOD	DPT	DCN	LUM	COL8A1	SFRP2	PDGFRA	COL1A1	FBLN1	LUM	SFRP2	THBS2	CTHRC1	THBS2	COL3A1	RARRES2	COL1A1	COL11A1	CREB3L1
**4**	SFRP4	FBLN1	SFRP4	IGF1	DCN	APOD	VCAN	C3	PDGFRA	CYP1B1	CTHRC1	MMP2	VCAN	PDGFRA	SFRP4	MMP2	SFRP2	GJB2	COL6A3	CTHRC1	COL1A2	ISLR	RP11-400N13.3
**5**	SFRP2	MMP2	TSHZ2	EFEMP1	FBLN1	MEG3	APOD	MMP2	COL10A1	MMP2	ITGBL1	LUM	SFRP4	COL1A2	DPT	COL1A1	COL10A1	SFRP2	LUM	SFRP2	COL6A3	MMP2	SFRP2
**6**	MMP2	SFRP4	HTRA3	PDGFRA	VCAN	OMD	FBLN1	APOD	SFRP4	VCAN	EFEMP1	COL1A2	COL1A2	EFEMP1	LUM	COL3A1	RARRES2	COL11A1	FN1	AEBP1	COL3A1	COL1A1	BASP1
**7**	RARRES1	SFRP2	FBLN1	OGN	FBLN5	ITGBL1	MMP2	EFEMP1	CTHRC1	CTHRC1	SFRP2	CTHRC1	MMP2	DCN	MEG3	ITGBL1	AEBP1	CCDC80	COL5A2	COL10A1	VCAN	COL1A2	PDPN
**8**	C3	RARRES1	MMP2	SFRP4	PDGFRA	PAPPA	COL6A3	MFAP4	APOD	LUM	FBLN5	DCN	SFRP2	CCDC80	EFEMP1	COL10A1	NBL1	NBL1	VCAN	NBL1	SFRP2	COL3A1	BNC2
**9**	MEG3	VCAN	COL6A3	VCAN	SFRP2	MRC2	COL1A1	FBLN1	MMP2	SFRP4	VCAN	CTSK	C1R	ISLR	VCAN	CTHRC1	CTSK	DCN	COL5A1	MMP2	MEG3	AEBP1	C5orf46
**10**	HTRA3	HTRA3	VCAN	CTSK	C3	LSAMP	COL1A2	SFRP4	PLXDC2	APOD	APOD	MFAP2	C1S	SFRP4	IGF1	LUM	VCAN	AEBP1	THBS2	CTSK	CTHRC1	MMP11	PLXDC2
**11**	FBLN1	ISLR	GPC3	COL1A2	MGP	CYP1B1	ISLR	CCDC80	VCAN	PLXDC2	COL8A1	APOD	APOD	COL6A3	FBLN5	SFRP2	CTGF	INHBA	SFRP2	THBS2	EFEMP1	THBS2	SPOCK1
**12**	MGP	COL6A3	CTGF	STEAP1	EFEMP1	COL10A1	COL10A1	RARRES1	BNC2	COL8A1	STEAP1	MATN3	CTSK	CYP1B1	SERPINE2	EFEMP1	COL8A1	LUM	CTHRC1	FBLN1	PDGFRA	COL12A1	ADM
**13**	DCN	SPON2	SFRP2	MMP2	OMD	COL8A1	CTHRC1	C1S	MRC2	FBLN1	COL1A2	MEG3	CCDC80	COL1A1	FBLN1	MRC2	THBS2	FBLN1	MMP2	VCAN	FBLN2	HTRA1	MMP2
**14**	CYP1B1	CYP1B1	C1S	CYP1B1	RP11-572C15.6	PDPN	CCDC80	VCAN	DPYSL3	OMD	PTGDS	ISLR	ISLR	APOD	SCN7A	COL6A3	MMP2	MMP2	COL10A1	CCDC80	LOX	MMP14	ARL4C
**15**	COL1A2	LXN	OMD	PTGDS	CCDC80	CXCL14	SFRP4	PTGDS	COL1A2	THBS2	ISLR	FBLN1	C3	CLDN11	LTBP2	PDPN	COL11A1	COL6A3	COL11A1	COL1A1	COL5A1	SFRP2	MEG3
**16**	MOXD1	SERPINF1	SPON2	FBLN1	IGF1	MMP23B	CTSK	C1R	FBLN5	FAP	LXN	COL11A1	EFEMP1	CTSK	APOD	VCAN	INHBA	OMD	AEBP1	S100A6	COL8A1	SULF1	GJA1
**17**	PTGDS	CTHRC1	C3	RARRES1	TSHZ2	ABCA9	COL3A1	CTSK	CREB3L1	MFAP2	OLFML3	COL3A1	MGP	FBLN1	PTGDS	DPYSL3	C1QTNF3	MEG3	COL12A1	COL8A1	LXN	LUM	VCAN
**18**	FBLN5	CTSK	F2R	DCN	ITM2A	LUM	S100A10	PDGFRA	COL3A1	COL6A3	THBS2	SFRP4	TSHZ2	MMP2	ISLR	LOX	SFRP4	COL8A1	DCN	TMSB10	MMP2	SDC1	FIBIN
**19**	PDGFRA	F2R	ANKH	COL3A1	SFRP4	PDGFRL	THBS2	SERPINF1	OMD	RARRES1	FBLN1	CXCL14	PDGFRA	COL3A1	PODN	GJA1	MATN3	ISLR	SPARC	C1S	S100A10	DCN	COL1A2
**20**	COL6A3	FBLN5	CTSK	MFAP5	RARRES1	STXBP6	HTRA1	MOXD1	RARRES1	EFEMP1	MGST1	ITGBL1	OMD	PTGDS	DCN	APOD	HTRA1	MMP11	TMSB10	COL1A2	FBLN1	MFAP5	ZFHX4
**21**	CTHRC1	COL1A2	C1R	FBLN5	COL8A1	SVEP1	SEMA3C	GPNMB	PODN	COL1A2	MEG3	COL6A3	FBLN5	FBLN2	MMP2	FAP	ITGBL1	MFAP5	MMP14	COL3A1	ISLR	VCAN	MFAP2
**22**	FAP	C7	IGFBP3	COL1A1	OGN	BICC1	FBLN2	RARRES2	LSAMP	ANKH	PDPN	VCAN	COL1A1	CTHRC1	MGP	MXRA5	MFAP5	PPAPDC1A	SDC1	HTRA1	FAP	COL6A3	MME
**23**	F2R	TMEM119	MOXD1	ISLR	C7	ABCA6	LRP1	FBLN5	THBS2	FNDC1	MFAP2	IGFL2	OGN	C3	C7	PODN	CXCL14	CTSK	POSTN	CD99	PPIC	GJB2	MFAP5
**24**	ISLR	EFEMP1	CTHRC1	C3	DPT	MFAP2	MRC2	ISLR	SVEP1	SPON2	DPT	RARRES2	SERPINF1	PLXDC2	MGST1	CXCL14	GJB2	MXRA5	FBLN1	ISLR	CYP1B1	GREM1	RAB3B
**25**	TIMP1	MOXD1	MEG3	MEG3	BICC1	BNC2	PDPN	ITGBL1	ITGBL1	PDPN	C3	FNDC1	CTGF	RARRES1	SPOCK1	COL8A1	IGFBP3	FNDC1	INHBA	SERPINF1	CREB3L1	TIMP2	ITGBL1
**26**	C7	CCDC80	PDGFRA	CTHRC1	CTHRC1	WNT5A	MXRA5	COL10A1	PTGDS	CTSK	MRC2	COL5A1	FBLN2	RP11-572C15.6	CCDC80	SFRP4	CCDC80	COL1A1	SERPINH1	TSC22D3	CCDC80	COL5A2	GJB2
**27**	PHLDA3	COL1A1	FBLN5	MOXD1	PODN	CST3	OMD	CYP1B1	FAP	MFAP5	PTGIS	OMD	COL6A3	THBS2	SLC19A2	SEMA3C	CD99	SDC1	MXRA5	FTL	MRC2	FAP	COL3A1
**28**	OMD	PLXDC2	ITM2A	COL6A3	COL6A3	SFRP2	ITGBL1	C7	LUM	HTRA3	MOXD1	CST4	PODN	C7	HTRA3	LRP1	C1S	VCAN	HTRA1	MFAP2	MFAP5	MFAP2	NTM
**29**	FBLN2	PDGFRA	COL1A2	MGP	CXCL14	MOXD1	RARRES2	PLXDC2	FBLN1	LRP1	HSD11B1	INHBA	LTBP2	LAMA2	MOXD1	NTM	LOXL1	GREM1	MMP11	ANXA2	THBS2	CTSK	PDLIM4
**30**	SCN7A	C3	PTGDS	C7	COL1A2	ZFHX4	FBN1	CTHRC1	SULF1	COL1A1	SFRP4	GJB2	ITGBL1	OLFML3	ITGBL1	C3	FIBIN	PDPN	ISLR	LAPTM4A	CTSK	COL5A1	CMTM8
**31**	EFEMP1	COL10A1	RARRES1	CILP	ISLR	RARRES1	PLXDC2	DPT	INHBA	DIO2	LSAMP	IGFBP3	C7	MFAP4	CTSK	COL11A1	FBLN1	FBLN2	MEG3	NNMT	MXRA5	PPAPDC1A	TANC2
**32**	COL10A1	C1S	OLFML3	COL8A1	MEG3	BOC	CXCL14	CLU	SEMA3C	DCN	PLXDC2	HTRA3	PLXDC2	SLIT2	FBLN2	UNC5B	PALLD	MFAP2	TIMP2	C1R	OGN	FN1	NT5E
**33**	SERPINF1	PODN	OGN	THBS2	CTSK	PODN	PDGFRA	NPC2	LOX	ALDH1A3	MFAP4	CST1	MOXD1	IGF1	CTHRC1	LOXL1	SDC1	C1QTNF3	FSTL1	RPL27A	RARRES2	TMEM158	TENM3
**34**	BNC2	LTBP2	ITGBL1	MRC2	HSD11B1	TMEM119	MATN3	MEG3	PDPN	LOX	OGN	FBLN2	TMEM119	LRP1	SVEP1	COL5A2	MFAP2	COL5A2	COL6A2	INHBA	FSTL1	POSTN	EPDR1
**35**	CTSK	NPC2	PTCH1	NR2F1	CTGF	PTGIS	COL8A1	RP11-572C15.6	IGF1	FBLN2	COL6A3	MFAP5	COL8A1	PDLIM3	MXRA5	SCARA3	ANXA2	CDH11	CTSK	NUPR1	COL5A2	ANTXR1	MYH10
**36**	MRC2	HSD11B1	COL8A1	ITGBL1	C1S	OGN	NBL1	MGP	LAMP5	OLFML3	SPON2	MXRA5	LRP1	LTBP2	STEAP1	COL8A2	APOD	LOX	MFAP2	LGALS1	LRP1	PLAU	LOX
**37**	TSHZ2	MGP	BOC	TSHZ2	COL10A1	FAP	TMEM119	COL6A3	FBLN2	TMEM119	COL1A1	THBS2	RARRES1	FBLN5	NEGR1	PTGDS	OMD	COL3A1	MFAP5	COL6A3	RARRES1	INHBA	COL8A1
**38**	LRP1	DPT	SERPINF1	STEAP2	SERPINE2	EFEMP1	HTRA3	LTBP2	OGN	MEG3	LTBP2	CTGF	BOC	MGP	C3	CCDC80	ISLR	PDGFRA	GAS1	COL11A1	FBLN5	GJA1	EVA1A
**39**	SLIT2	COL3A1	MGP	MFAP2	SERPINF1	LAMA2	FAP	AEBP1	PTGFRN	FGF7	PODN	MRC2	CYBRD1	MEG3	RARRES1	ALDH1A3	SLC6A6	F13A1	LRRC15	OMD	FBN1	LGALS1	MXRA5
**40**	COL1A1	OMD	IGFBP6	PDGFRL	LTBP2	GSTM5	MEG3	PDPN	GAS7	TMSB10	DCN	GJA1	LXN	BICC1	RP11-572C15.6	FGFR1	CYR61	FRMD6	COL8A2	CD55	GAS1	PTK7	BICC1
**41**	SVEP1	MFAP4	TIMP1	PDPN	NEGR1	IGF1	FNDC1	COL1A1	CXCL14	CXCL14	CTSK	FAP	LOX	MRC2	PLXDC2	BOC	COL1A2	APOD	FBLN2	PDPN	BNC2	CD99	C1orf198
**42**	THBS2	TSHZ2	MFAP4	PLXDC2	MOXD1	F2R	NTM	COL8A1	COL11A1	ITGBL1	SPOCK1	SPON2	HTRA3	FGFR1	LRP1	PLXDC2	MMP11	COL1A2	GREM1	LOXL1	PLOD2	NBL1	INHBA
**43**	TMEM119	COL8A1	CLEC11A	OMD	C1R	F3	DPYSL3	S100A13	FNDC1	IGFBP3	COL3A1	BICC1	RP11-572C15.6	ABL2	ABI3BP	MFAP2	MEG3	DIO2	APOD	FIBIN	PDPN	NTM	PDGFC
**44**	IGFBP3	STEAP1	INHBA	MMP23B	FGF7	MMP2	SLC6A6	OGN	HTRA3	CDH11	RARRES1	EMP1	CYP1B1	MXRA5	FAP	MFAP5	PLXDC2	CD55	COL8A1	NTM	MMP23B	RARRES2	FBLN2
**45**	C1S	PTGDS	MXRA8	LXN	COL1A1	SFRP4	CYP1B1	MMP23B	MOXD1	IGF1	SVEP1	FGF7	CTHRC1	RGS2	COL6A3	TMSB10	FBLN2	HTRA1	CD99	S100A10	LTBP2	FBLN1	B4GALT1

#### Further demonstration of the continuity of the transition

**Fig 1:**
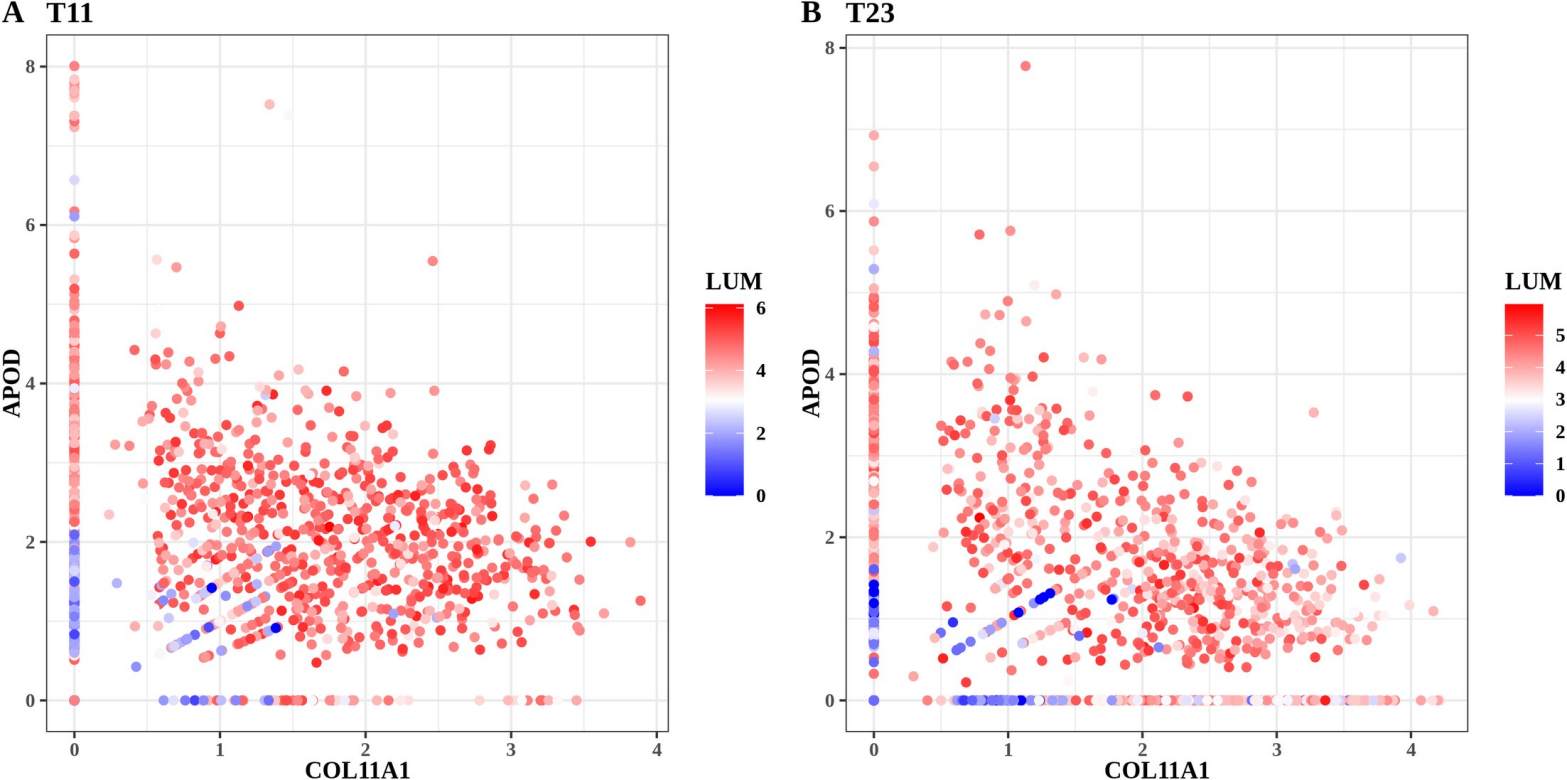
**Scatter plots for fibroblast-rich samples for patients (A) T11 and (B) T23.** Each dot represents a mesenchymal cell identified in the sample. The x- and y-axis denote the expression levels of *COL11A1* and *APOD*, respectively. Dots are colored for the expression of fibroblastic marker *LUM*. The expression unit is the normalized log-transformed value from the count matrix (Materials and methods).

As an additional confirmation of the continuity of the transition (as opposed to the presence of a mixture of distinct fibroblastic subtypes), Fig 1 shows scatter plots for genes *APOD* and *COL11A1*, color-coded for the expression of fibroblastic marker *LUM*, of the mesenchymal cells in two fibroblast-rich samples T11 and T23. The presence of cells covering the full range from the upper-left to the bottom-right sides of the plots, including the intermediate stages in which cells co-express both markers, demonstrates the presence in each sample of cells representing the continuously varying transition from ASCs to *COL11A1*-expressing CAFs.

To further investigate the continuous transition, we partitioned the 34 pancreatic samples into three groups. Group 1 includes the eleven normal samples (N1 to N11). For tumor samples, we divided the rearranged samples in Table 5 into two groups (Group 2 and Group 3). Group 2 contains all samples to the left of and including T22, so that *APOD* is ranked before *COL11A1* in the attractors of that Group, representing a relatively earlier stage of this transition. We then applied the consensus version of the attractor finding algorithm (Materials and Methods) and identified the signatures representing the main state of the fibroblasts for each of the above three sample groups (Table 6). Although there are many shared genes, the groups have distinct gene rankings. Group 1 (normal samples) contains many adipose-related genes, consistent with Table 2. Group 3 contains, in addition to *COL11A1*, many among the other CAF genes, such as *THBS2*, *INHBA*, *AEBP1*, *MFAP5* and *COL10A1*. Group 2 displays an intermediate state, including markers of both ASCs as well as CAFs.

**Table 6:** Top 30 genes of the consensus attractors for three different PDAC sample groups. Group1: normal samples; Group 3: T11, T21, T23, T9, T16, T17, T8; Group 2: other tumor samples.

Rank	Group1	Group2	Group3	Rank (cont’d)	Group1	Group2	Group3
**1**	LUM	LUM	COL1A1	**16**	PDGFRA	CYP1B1	MMP2
**2**	DCN	SFRP2	COL1A2	**17**	SRPX	FBLN5	DCN
**3**	FBLN1	APOD	COL3A1	**18**	COL6A3	MEG3	SFRP2
**4**	C7	SFRP4	FN1	**19**	ADH1B	COL1A1	TMSB10
**5**	APOD	MMP2	COL5A2	**20**	CFD	C3	POSTN
**6**	PTGDS	VCAN	COL5A1	**21**	OLFML3	RARRES1	MXRA5
**7**	SFRP2	PDGFRA	COL6A3	**22**	SERPINF1	CCDC80	COL6A2
**8**	C1S	FBLN1	COL11A1	**23**	MMP2	MOXD1	ISLR
**9**	CCDC80	DCN	CTHRC1	**24**	CST3	PLXDC2	AEBP1
**10**	MGP	EFEMP1	THBS2	**25**	SEPP1	HTRA3	MEG3
**11**	DPT	CTHRC1	VCAN	**26**	ABCA8	COL10A1	MFAP5
**12**	CXCL12	ISLR	COL10A1	**27**	COL1A2	COL8A1	SERPINH1
**13**	C1R	COL6A3	LUM	**28**	LAMB1	ITGBL1	MMP14
**14**	FBLN5	COL1A2	SPARC	**29**	SVEP1	OMD	MFAP2
**15**	C3	CTSK	COL12A1	**30**	MEG3	PTGDS	INHBA

To find potential critical genes at the initiation phase of the cellular transition, we focused on the first tumor samples (with highest *APOD* ranking) in Table 5, so we can compare them with those of the normal ASCs.

We observe that gene *SFRP4* stands out, as it appears for the first time remarkably among the top genes in all the first samples T2, T13, T14, T19, ranked 4th, 6th, 4th 8th, respectively. This suggests that the Wnt pathway is involved in the initiation of the cellular transition, because *SFRP4* is a Wnt pathway regulator whose expression has been found associated with various cancer types [25,26]. Interestingly, *SFRP4* disappears from the list of the attractors, indicating that it is downregulated in the final stage of the transition.

It is also known that gene *RARRES1* (aka TIG1) plays an important role in regulating the proliferation and differentiation of ASCs [27]. Consistently, Table 6 reveals that *RARRES1* appears for the first time in the attractors of the initial tumor samples. Just like *SFRP4*, *RARRES1* is downregulated in the final stage, related to the fact that it has been suggested as a tumor suppressor [28,29].

We also performed differential expression (DE) analysis comparing the normal samples with the first samples (T2, T13, T14, T19) of Table 6 (Materials and Methods; S2 Table). The results of such DE analysis represent the full population of fibroblasts and not necessarily reflect the expression changes in the particular cells undergoing the ASC to *COL11A1* expressing CAF transition. Gene *CFD* was found to be most downregulated, consistent with the expected downregulation of adipose-related genes as they differentiate into fibroblasts. Genes *SFRP4* and *RARRES1* are upregulated consistent with their appearance in the attractors.

On the other hand, the top upregulated gene is phospholipase A2 group IIA (*PLA2G2A*), which is not among the top genes of any attractors we identified, indicating that it is not expressed by cells undergoing the ASC to *COL11A1*-expressing CAF transition. It probably still plays, however, an important related parallel role and many previous studies referred to its effects on prognosis of multiple cancer types [30–32]. The PLA2G2A protein is a member of a family of enzymes catalyzing the hydrolysis of phospholipids into free fatty acid. We hypothesize that this process leads to fatty acid oxidation, which may facilitate metastatic progression. Indeed, it has been recognized that fatty acid oxidation is associated with the final *COL11A1*-expressing stage of the transition [33]. These results suggest that lipid metabolic reprogramming plays an important role in the metastasis-associated biological mechanism [34], by potentially providing energy for the metastasizing tumor cells.

#### Validation with trajectory inference

We independently applied trajectory inference (TI) analysis on the PDAC fibroblasts by using the Slingshot [35] method in an unsupervised manner. We first performed unsupervised clustering on the identified fibroblasts (Materials and Methods), resulting in four subgroups X1, X2, X3, X4 (S1A Fig) with the top differentially expressed genes shown in S1B Fig. One of these clusters (X4) was discarded from further TI analysis, because it mainly expressed the IL1 CAF marker *HAS1* (Hyaluronan Synthase 1), which is not expressed by either ASCs or *COL11A1*-expressing CAFs (and does not appear at all in S1 Table), and contained only 3% of fibroblasts resulting almost exclusively from patient T11 (S1C Fig).

**Fig 2:**
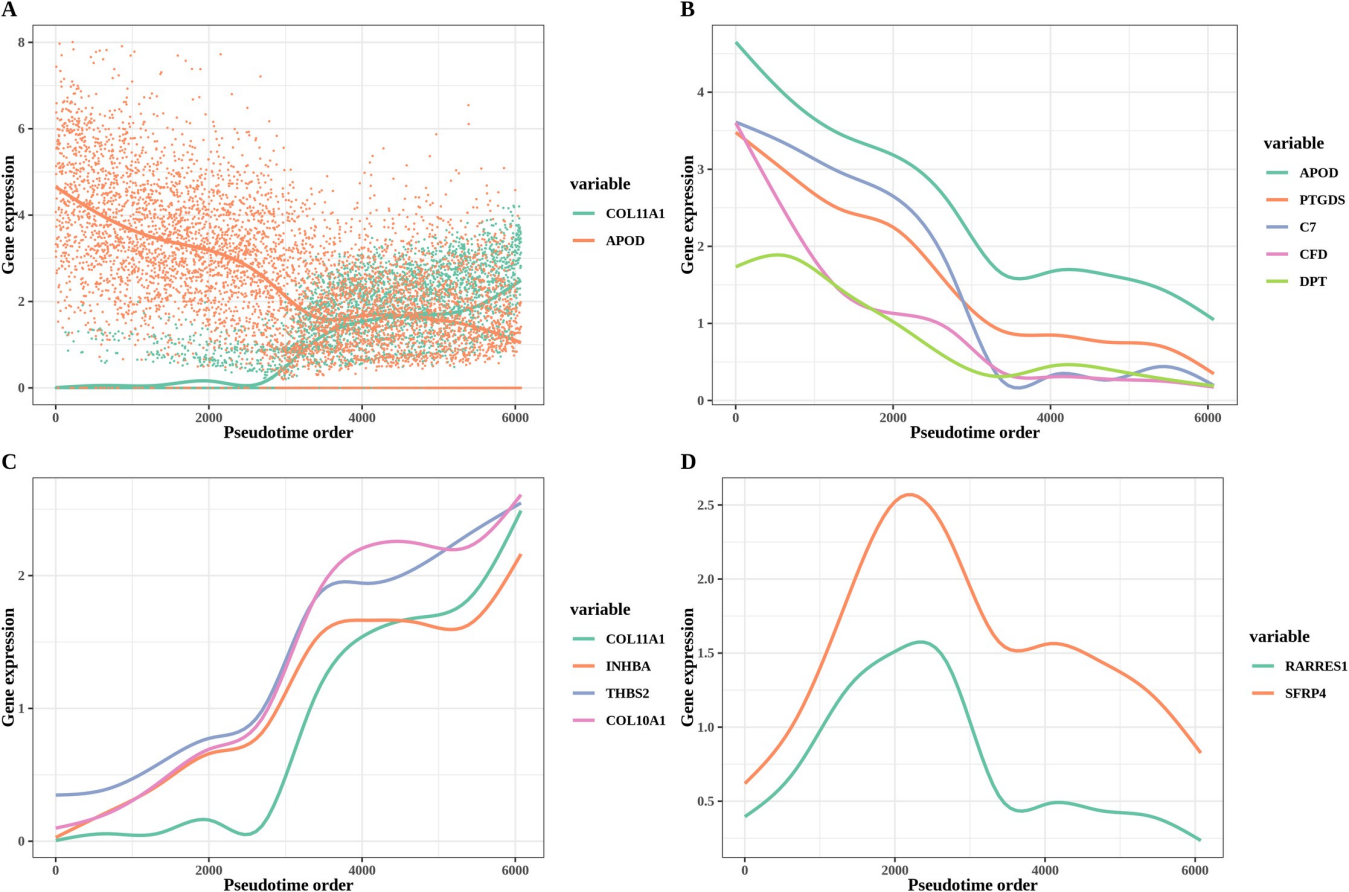
Trajectory analysis of PDAC. **A.** GAM fit to pseudotime ordered expression data to visualize the trend of gene expressions. **B.** Expression of adipose-related genes along the transition lineage. The x axis shows the cell orders and the y axis shows the normalized read count. **C.** Expression of *COL11A1*-associated genes along the transition lineage. **D.** Expression of *RARRES1* and *SFRP4* genes along the transition lineage.

As seen from the list of top differentially expressed genes of each cluster, X1 contains CAF genes top ranked (including *MMP11*, *COL11A1*, *THBS2*, *INHBA*), X2 has *RARRES1* at the top, and X3 has ASC genes top ranked, including *DPT*, *C7*, *CXCL12* and *CFD*. Consistently, S2A and S2B Fig show the single trajectory path resulting from TI analysis, where X3 is the starting point and X1 is the end point of the trajectory, while X2 (highly expressing *RARRES1*), is an intermediate point, thus validating the continuous ASC to *COL11A1*-expressing CAF transition. The orderings of patient groups and sample identity (S2C and S2D Fig) are also consistent with our previous findings based on attractor analysis. S3 Table shows the top 100 genes with zero *P* value, ranked by their variances, resulting from pseudotime-based differential gene expression analysis (Materials and Methods). We can clearly identify as top-ranked several ASC genes, as well as CAF genes, while some general fibroblastic markers, such as *DCN*, are missing, consistent with the continuity of the ASC to *COL11A1*-expressing CAF transition. We then used a generalized additive model (GAM) fit to pseudotime-ordered expression data to visualize the trend of gene expressions (Fig 2A).

There was a prominent difference between adipose-related genes and *COL11A1*-associated genes. The expression of the adipose-related genes steadily fell across the process (Fig 2B), while the expression of *COL11A1*-associated genes gradually increased (Fig 2C). There is a significant negative correlation between these two groups of genes, e.g., *COL11A1* (the last among those genes to increase its expression) was exclusively overexpressed in the mature CAFs, which did not express *C7*. Of particular interest, genes *SFRP4* and *RARRES1* (Fig 2D) increased consistently at the beginning and then decreased after reaching a peak, suggesting that they may play important roles in the differentiation path.

### Validation in other cancer types

Next, we validated the ASC to *COL11A1*-expressing CAF transition in other solid cancer types. Although we could not find currently available datasets as rich as the PDAC dataset, we selected those containing a large (at least 100) number of fibroblasts and separately analyzed each of them, obtaining consistent results. Specifically, we used four scRNA-seq datasets from head and neck cancer (HNSCC) [36], ovarian cancer[37], lung cancer [38] and breast cancer [39].

The *COL11A1*-expressing CAF signature has been confirmed to be a pan-cancer signature [40–42]. Therefore, the most important validation task would be to confirm the existence of the *APOD*/*CFD*/*CXCL12*/*MGP*/*PTGDS*-expressing ASCs as the starting point of the transition, and to also confirm that some samples are at an intermediate stage, expressing genes such as *SFRP4*, *RARRES1* and *THBS2*, in addition to the core ASC genes, demonstrating that they are at an intermediate stage of the transition.

#### Head and neck squamous cell carcinoma

For the HNSCC dataset, the authors of the paper presenting the data [36] reported that the cancer-associated fibroblasts in the dataset can be partitioned into two subsets, which they name CAF1 and CAF2. In S5 Table of that paper, the top three differentially expressed genes of the CAF2 group are *CFD*, *APOD* and *CXCL12*, while the full gene list for CAF2 presented in the same S5 Table also includes genes *MGP*, *C3*, *C7*, *DPT*, *PTGDS*. This strongly suggests that the partitioning used in the paper was influenced by the presence of an ASC cell subpopulation, identical, or at least very similar to, those discovered in the PDAC. Similarly, the list of differentially expressed genes for CAF1 in S5 Table includes genes *INHBA*, *THBS2*, *CTHRC1*, *POSTN*, *MMP11*, *COL5A2*, *COL12A1*, suggesting that the identified CAF1 subpopulation was influenced by the presence of differentiated CAFs, which would eventually express *COL11A1*. Finally, gene *RARRES1* also appears among the list of CAF2 genes, suggesting that it was captured among cells which had started the process of ASC to *COL11A1*-expressing CAF transition.

In our independent analysis, we performed clustering identifying 1,026 fibroblasts from all available cells (S3A Fig; Materials and Methods). There were two fibroblastic clusters (X7 and X9) expressing CAF associated genes (*COL11A1*, *COL12A1*, *MMP11*, *INHBA*, *THBS2*, *COL10A1*, *COL8A1*, *FN1*) and ASC associated genes (*APOD*, *C7*, *PTGDS*), respectively (S4 Table), which confirmed the presence of these two populations in HNSCC.

Among the individual patients, we found that the most prominent case is sample HNSCC28, which contains a rich set of cells undergoing differentiation. Applying the attractor finding algorithm on the fibroblasts of that sample (S5 Table) resulted in genes *LUM*, *APOD*, *COL6A3*, *PDGFRA*, *DCN*, and *CFD* being among the top-ranked, revealing that it represents an ASC population. Furthermore, the presence of genes *THBS2*, *MFAP5* and *VCAN* in the same attractor reveals that these cells have already started undergoing the transition.

#### Ovarian cancer

For the ovarian dataset, the clustering results showed two clusters (X6 and X9) expressing *COL11A1*-associated genes and ASC-associated genes, respectively (S3B Fig, S6 Table; Materials and Methods). Among the individual patients, we found that the ones validating our hypotheses most are HG2F and LG2, both of whose datasets, consistently, contain cells from the fatty omental tissue. S5 Table includes the corresponding two attractors identified in the cells of each patient. Among the top ranked genes for HG2F are *DCN*, *LUM*, *C1S*, *C7*, and *C3*, but also *RARRES1*, suggesting that they represent fibroblasts undergoing the transition, while the LG2-based attractor contains highly ranked all three genes *COL11A1*, *INHBA*, *THBS2*.

#### Lung cancer

The dataset contains a large number (> 50,000) of cells, but we only classified ~2% (= 1,346) among them as mesenchymal cells, including fibroblasts and pericytes (Materials and Methods). Among those cells, there were two fibroblastic clusters (X1 and X2) expressing related genes (*COL11A1*, *COL12A1*, *MMP11*, *INHBA*, *THBS2*, *COL10A1*, *COL8A1*, *FN1*) and ASC related genes (*APOD*, *C7*, *PTGDS*), respectively (S3C Fig, S7 Table). The presence of the transition is evident by the attractors identified in the mesenchymal cells for patients 4 and 3 (S5 Table). The former prominently contains genes *CFD*, *PTGDS* and *C7*, while the latter contains *THBS2*, *COL10A1* and *INHBA*.

#### Breast cancer

The size of the breast cancer dataset is small (~1,500 cells in total), and 169 cells among them were classified as mesenchymal (Materials and Methods). By further clustering these cells, we identified ASCs (X1) and *COL11A1*-expressing CAFs (X3) (S3D Fig, S8 Table). ASC related genes (*APOD*, *MFAP4*, *CFD*) were identified in X1, while CAF-related genes (*COL10A1*, *COL11A1*, *MMP11*, *INHBA*, *FN1*, *THBS2*, *AEBP1*, *COL12A1*) are among the top 15 of X3. Patients PT089 and PT039 contain the highest proportions (>50%) of the ASC and *COL11A1*-expressing CAF subpopulations, respectively, and we found consistent results in their attractors (S5 Table), as the former contains *C1S*, *C1R*, *CXCL12*, *PTGDS*, *C3*, while the latter contains *THBS2*, *COL11A1*, *COL10A1*, at top-ranked positions.

### Potential therapeutic targets inhibiting the invasiveness-associated transition

This work provides opportunities for identifying therapeutic targets inhibiting the cellular transition. For example, targeting of gene *MFAP5* was recently found to enhance chemosensitivity in ovarian and pancreatic cancers [43]. Specifically, the author states that “*MFAP5* blockade suppresses fibrosis through downregulating of fibrosis-related genes such as *COL11A1*.” Consistently, we found *MFAP5* as one of the most highly associated genes with *COL11A1* (Table 4).

As mentioned earlier, genes *SFRP4* and *RARRES1* are transiently expressed in Group 2 of Table 6, suggesting that they can be investigated for inhibiting the cellular transition.

Of particular interest as potential drivers are noncoding RNAs due to their typical regulatory role. Because the expression of these genes is not accurately captured by scRNA-seq technology, we did a thorough analysis of the full set of The Cancer Genome Atlas (TCGA) pan-cancer data. For the RNA sequencing and miRNA sequencing dataset of each cancer type, we removed the genes in which more than 50% of the samples have zero counts. Then we performed quantile normalization using the limma package [44] (v3.40.6) on log2 transformed counts. In each of the 33 cancer types, we ranked all protein-coding genes in terms of the association (using the metric of mutual information) of their expression with that of gene *COL11A1*. We excluded the 11 cancer types (*LGG*, *SKCM*, *SARC*, *LAML*, *PCPG*, *GBM*, *TGCT*, *THYM*, *ACC*, *UVM*, *UCS*) in which neither *THBS2* nor *INHBA* was among the 50 top-ranked genes, because of the absence of significant amounts of *COL11A1*-expressing CAFs in those samples (1st sheet in S9 Table). In each of the remaining 22 cancer types, we then ranked all long noncoding RNAs (lncRNAs) and microRNAs (miRNAs) in terms of their association with *COL11A1* (2nd and 3rd sheets in S9 Table). Finally, we did pan-cancer sorting of all lncRNAs and miRNAs in terms of the median rank of all lncRNAs and miRNAs (4th sheet in S9 Table).

We found that *LINC01614* represents a particularly promising therapeutic target. It had a perfect score of 1 in the pan-cancer sorting list, being strikingly the top-ranked gene in 14 (BRCA, UCEC, KIRC, HNSC, LUAD, LUAD, LUSC, OV, STAD, ESCA, PAAD, MESO, DLBC, CHOL) out of the 22 cancer types (2nd sheet in S9 Table). In fact, the association of *LINC01614* was even higher than that of marker protein-coding gene *INHBA*. The pan-cancer consensus ranking of protein-coding genes in terms of *LINC01614* (5th sheet in S9 Table) corresponds precisely to the *COL11A1*-expressing CAF signature. These rankings, in which marker genes unique to the original and intermediate stages are missing, indicate that *LINC01614* is involved in the very final stage of the creation of the *COL11A1*-expressing CAFs. Therefore, we hypothesize that therapeutics targeting *LINC01614* specifically in patients’ CAFs may inhibit the final metastasis-facilitating stage of the transition.

We also found that the three top-ranked miRNAs were *miR-199a-1*, *miR-199b*, *miR-199a-2*. The associated *miR-214* is also very highly ranked (3rd sheet in S9 Table).

## Discussion

Our results indicate that the cancer invasiveness-associated *COL11A1*-expressing CAFs are produced as a result of the interaction of tumor cells with the adipose microenvironment. Therefore, one contribution of our work is that it provides a potential explanation to the well-known fact that adipose tissue contributes to the development and progression of cancer [45–47].

Another contribution is that it precisely identifies the ASC population, as evidenced by the consistent presence of its marker genes among the top-ranked attractor genes in each of the eleven columns of Table 2. The identification of those particular marker genes (*APOD* prominent among them) cannot be due to chance, because these were eleven totally independent unbiased experiments, and also because the attractor algorithm applied on the SVF of normal adipose tissue in another independent dataset identified precisely the same genes. This finding could not have been achieved with traditional methods.

There is consensus agreement that CAFs are a promising potential target for optimizing therapeutic strategies against cancer, but such developments are restricted by our current limitations in our understanding of the origin of CAFs and heterogeneity in CAF function [48]. Therefore, there is an urgent need to enhance our understanding of those matters. Our results provide clarity on one important particular component (out of several) of the heterogeneous fibroblast tumor microenvironment. To avoid potential erroneous conclusions after applying bioinformatics algorithms, single-cell data analysis provides an unprecedented capability to validate results, including those resulting from the attractor algorithm, by “seeing” individual cells in color-coded scatter plots, such as the one shown in Fig 1, observing and confirming the presence or absence of distinct populations characterized by the combined presence of particular marker genes.

In particular, there are several published papers relying on the application of clustering algorithms following dimensionality reduction on the particular datasets they use, and concluding that there exist a number of distinct and mutually exclusive CAF subpopulations. These reported fibroblastic subpopulations occasionally have gene expression profiles that are conflicting with each other in significant ways among these publications. Examples include the hC1 and hC0 clusters in [49], the C9 and C10 clusters in [42], the CAF2 and CAF1 clusters in [36], the iCAF and myCAF clusters in [50,51] and the iCAF an mCAF clusters in [52]. A review of such results in pancreatic cancer appears in [53].

As an example of conflicting results, the “iCAFs” identified in [52] have significant differences from those identified in other papers and are, in fact, identical to the normal ASCs (Fig 3B of [52]) identified in this paper, as evidenced by the list of its differentially expressed genes (*PTGDS*, *LUM*, *CFD*, *FBLN1*, *APOD*, *DCN*, *CXCL14*, *SFRP2*, *MMP2*, all of which appear in Table 3, further validating the ASC signature. Therefore, this identified cluster contains mainly normal cells at the origin of the transition, which should not even be called CAFs.

Similarly, a recent single-cell data analysis [54] identified two clusters “touching” each other in a UMAP plot (Fig 2A of [54]), C0 and C3, which are precisely the two endpoints of the ASC to *COL11A1*-expressing CAF transition. Indeed, as identified in Table S6-1 of [54], C0 cluster has the marker genes *APOD*, *PTGDS*, *C7*, *C3*, *MGP*… which the attractor algorithm had identified and validated in this paper. On the other hand, the marker genes of cluster C3 are precisely those of the *COL11A1*-expressing CAFs, in which all three genes *COL11A1*, *INHBA* and *THBS2* are top-ranked (because the metastatic process was already underway). Importantly, as shown in Fig 2B of [54], the ASC marker genes *APOD* and *PTGDS* (top ranked in C0 and unrelated to CAFs) are significantly expressed even in the *COL11A1*-expressing cluster C3 of the paper, providing further evidence of the presence of intermediate states consistent with the transition–and the separating line between C0 and C3 in the diagram is not generated by any biologically reliable manner, consistent with the continuity.

On the other hand, our work is consistent with, and complementary to the results of [49] focusing on the immunotherapy response, in which the presence of the “TGF-beta CAFs” was inferred by an 11-gene signature consisting of *MMP11*, *COL11A1*, *C1QTNF3*, *CTHRC1*, *COL12A1*, *COL10A1*, *COL5A2*, *THBS2*, *AEBP1*, *LRRC15*, *ITGA11*. This population apparently represents the *COL11A1*-expressing CAF endpoint of the transition, and gene *LRRC15* was selected as the representative gene based on the fact that it was found to be the most differentially expressed gene between CAFs and normal tissue fibroblasts in mouse models. Indeed, *LRRC15* is a key member of the *COL11A1*-expressing CAF signature (Table 4 of [1]) and we also found that *COL11A1* is the highest associated gene to *LRRC15* in the Group 3 PDAC patients.

In our work we used a detailed gene association-based scrutiny of all our results, including numerous color-coded scatter plots, rather than blindly accepting clustering results. We believe that this nontraditional computational methodology, when used on rich single-cell data, represents a paradigm shift in which systems biology alone can be trusted, by itself, for producing reliable results. We hope that our results will give rise to testable hypotheses that could eventually lead to the development of pan-cancer therapeutics inhibiting the ASC to *COL11A1*-expressing CAF transition.

## Materials and methods

### Datasets availability

The pancreatic dataset [19] was downloaded from the Genome Sequence Archive with accession number CRA001160. The four validation datasets of other cancer types are also publicly available: HNSCC [36] (GSE103322), ovarian [37] (GSE118828), lung cancer [38] (E-MTAB-6149 and E-MTAB-6653), breast cancer [39] (GSE118389). We excluded samples from lymph nodes. The numbers of patients included in these datasets are 35 (PDAC), 18 (HNSCC), 9 (ovarian), 5 (lung), and 6 (breast).

### Data processing and cell identification

We selected the Seurat R toolkit [55] for data processing and cell identification. Seurat implements the entire clustering workflow and has an advantage in speed and scalability to analyze large datasets [56]. We applied the Seurat (v3.1.4) to process the gene expression matrix and characterize the cell type identity for each scRNA-seq dataset. The count matrix was normalized and log transformed by using the NormalizeData function. We selected the 2,000 most variable features and then performed principal component analysis (PCA) followed by applying an unsupervised graph-based clustering approach. We used default parameter settings in all the above steps except that the resolution parameter in the FindCluster function is set to 1.0 to increase the granularity of downstream clustering. To identify differentially expressed genes for each cluster, we used the FindMarkers function. To characterize the identity of mesenchymal cells in each dataset, we made use of the expression of known markers: *LUM*, *DCN*, *COL1A1* for fibroblasts, and *RGS5*, *ACTA2*, *PDGFRB and ADIRF* for pericytes.

For the smaller-size datasets (ovarian, breast), we performed clustering once on all cells for mesenchymal cell identification. For datasets of larger size (PDAC, HNSCC, lung), we applied ‘two-step clustering’ to ensure accuracy: The first step was initial clustering within individual samples. Then we excluded samples with very few (< 20) detected fibroblasts and pooled the mesenchymal cells of the remaining samples together for a second clustering, which resulted in the final set of mesenchymal cells for the dataset. For the PDAC dataset, we included an additional step to remove low-quality cells, by retaining cells for which at least one of the corresponding markers had expression levels ≥ 3.

### Mutual information

Mutual information (MI) is a general measure of the association between two random variables [57]. We used a spline based estimator [58] to estimate MI values and normalized so the maximum possible value is 1. The MI value is clipped to zero if the Pearson correlation between the two variables is negative. The details of the estimation method are described in the paper introducing the attractor algorithm [20]. We used the getMI or getAllMIWz function implemented in the cafr R package with parameter negateMI = TRUE.

### Attractor-based analysis

The attractor algorithm was first proposed for identifying co-expression signatures from bulk expression values in samples [20]. In this study, we use the attractor algorithm for the first time for the purpose of scrutinizing cell populations in single-cell data. Compared to conventional single-cell methods, the attractor algorithm features the unique capability of discovering precise profiles of cell populations, which other methods cannot achieve (see Discussion).

Briefly, the algorithm iteratively finds mutually associated genes from an expression matrix, converging to the core of the co-expression mechanism. The association measure used is the normalized mutual information (as described above), which captures the general relationships (including nonlinear effects) between variables. Using the expression vector corresponding to a seed gene as input, the algorithm converges to an “attractor” in the form of a list of ranked genes, together with scores (ranging from 0 to 1) for each of these genes measuring the strength of the membership of that gene in the signature. It has a characteristic property that using different “attractee” genes belonging to a co-expression signature as seeds leads to the identical attractor.

The attractor algorithm had previously been used to find co-expression signatures in bulk gene expression data, in which case a converged attractor could represent a mixture of contributions from distinct cell subpopulations. When using single-cell data, however, the characteristic genes of particular distinct subpopulations will have high expression values only in the cells from those subpopulations and low values in other cells. These genes will have pairwise positive and large correlations, and therefore they will be highly ranked in attractor signatures representing such individual subpopulations. On the other hand, two characteristic marker genes belonging to two different distinct subpopulations will have reverse-associated expression values across those cells, which will contribute negatively to the overall correlation between these two genes. Only if two genes are co-expressed across individual cells will they appear highly ranked in the same attractor.

For single dataset, we applied the attractor finding algorithm using the findAttractor function implemented in the cafr (v0.312) R package [20] with the general fibroblastic marker gene *LUM* as seed. Identical results in all samples will be found, with very rare exceptions, if other fibroblastic markers, such as *DCN*, are used. The exponent (*a*) was set to different values for scRNA-seq datasets profiled from different protocols. For the analysis of UMI based (e.g. 10x) and full-length-based (e.g. Smart-seq2) datasets, we used *a* = 3 and *a* = 5, respectively. To find the consensus attractor for multiple datasets, we applied the consensus version of the attractor finding algorithm as described in [59]. In the consensus version, the association measures between genes are evaluated as the weighted median of the corresponding measures taken from the individual datasets. The weights are proportional to the number of samples included in each individual dataset in log scale.

### Trajectory inference (TI) analysis

We selected the Slingshot [35] method for TI analysis, based on its robustness and suggestions made by the benchmarking pipeline dynverse [18]. We used the raw counts as input and followed the Slingshot lineage analysis workflow (v1.4.0). To begin this process, Slingshot chose robustly expressed genes if it has at least 10 cells that have at least 1 read for each. After gene filtering, we proceeded to full quantile normalization. Following diffusion map dimensionality reduction, Gaussian mixture modelling was performed to classify cells, where the number of clusters in the Mclust function was set to 3 based on the fact that there were three clusters in our previous Seurat clustering results. The final step of lineage inference analysis used the slingshot wrapper function in an unsupervised manner. A cluster based minimum spanning tree was subjected to describe the lineage. After analyzing the global lineage structure, we fitted a generalized additive model (GAM) for pseudotime and computed *P* values. Genes were ranked by *P* values and variances. After running Slingshot, we identified genes whose expression values significantly vary over the derived pseudotime by using a GAM, allowing us to detect non-linear patterns in gene expression.

### Statistical analysis

#### *P* value evaluation for overlapping genes from different sets

We applied the hypergeometric test for evaluating the significance of genes shared by different sets. If there are two sets to compare, we used the phyper R function. If there are more than two sets to compare, we employed the multi-set intersection test [5] by applying the cpsets function implemented in the SuperExactTest R package. Regarding the background universe size of genes, we used the total number of genes analyzed in the specific expression matrix. In the case of comparing sets coming from different studies, we used 20,000 as the universe size.

#### Differential expression analysis

We used a Wilcoxon Rank Sum test by applying the FindMarkers function in Seurat to identify the differentially expressed (DE) genes between fibroblasts of different groups. DE genes with |log fold change| > 0.25 and Bonferroni adjusted *P* value < 0.1 are considered as significant. The positive and negative DE genes are ranked separately in terms of the absolute values of their log fold-change.

## Supporting information

S1 Fig Overview of the PDAC fibroblasts. **A.** 6,267 fibroblasts originated from 11 control pancreases and 23 tumor samples were petitioned into four groups X1-X4. Fractions of the fibroblasts were: 45%, 38%, 14%, and 3%. **B.** Table showing the top 20 DE genes for each cluster. **C.** Bar plots presenting the numbers of cells captured for each cluster.
(TIF)

S2 Fig Trajectory analysis of 6,075 fibroblasts in PDAC dataset. **A.** Colors coded for pseudotime changing, red presenting the beginning of differentiation and blue presenting the end. **B.** Color-coded trajectory analysis of fibroblasts for annotated three clusters. **C.** Color-coded trajectory analysis of fibroblasts for group information. **D**. Color-coded trajectory analysis of fibroblasts for sample identity.
(TIF)

S3 Fig Unsupervised clustering of four datasets from HNSCC, ovarian cancer, lung cancer and breast cancer. **A.** t-SNE embedding of the whole HNSCC dataset. **B.** t-SNE embedding of the whole ovarian cancer dataset. **C.** t-SNE embedding of the mesenchymal cells from lung cancer dataset. **D.** t-SNE embedding of the mesenchymal cells from breast cancer dataset.
(TIF)

S1 Table *LUM*-seeded attractors (top 100 genes) identified in each PDAC sample. (XLSX)

S2 Table Differentially expressed genes comparing normal pancreatic samples against four PDAC samples at the initial phase of the transition. (XLSX)

S3 Table Top 100 genes of temporally expressed genes on the pseudotime variable. (XLSX)

S4 Table Differentially expressed genes among different clusters of HNSCC dataset. (XLSX)

S5 Table *LUM*-seeded attractors (top 100 genes) from validating samples of other cancer types. (XLSX)

S6 Table Differentially expressed genes among different clusters of ovarian cancer dataset. (XLSX)

S7 Table Differentially expressed genes among different clusters of mesenchymal cells from lung cancer dataset. (XLSX)

S8 Table Differentially expressed genes among different clusters of stromal cells from breast cancer dataset. (XLSX)

S9 Table Ranked genes lists in terms of their association (mutual information) with gene *COL11A1* by using the full set of pan-cancer TCGA bulk RNA-seq data. (XLSX)
