# Supplementary figures and images for: Single-cell analysis reveals the pan-cancer invasiveness-associated transition of adipose-derived stromal cells into COL11A1-expressing cancer-associated fibroblasts
Source: PLoS Comput Biol. 2021 Jul 20;17(7):e1009228. doi: 10.1371/journal.pcbi.1009228 (PMC8323949; doi:10.1371/journal.pcbi.1009228)

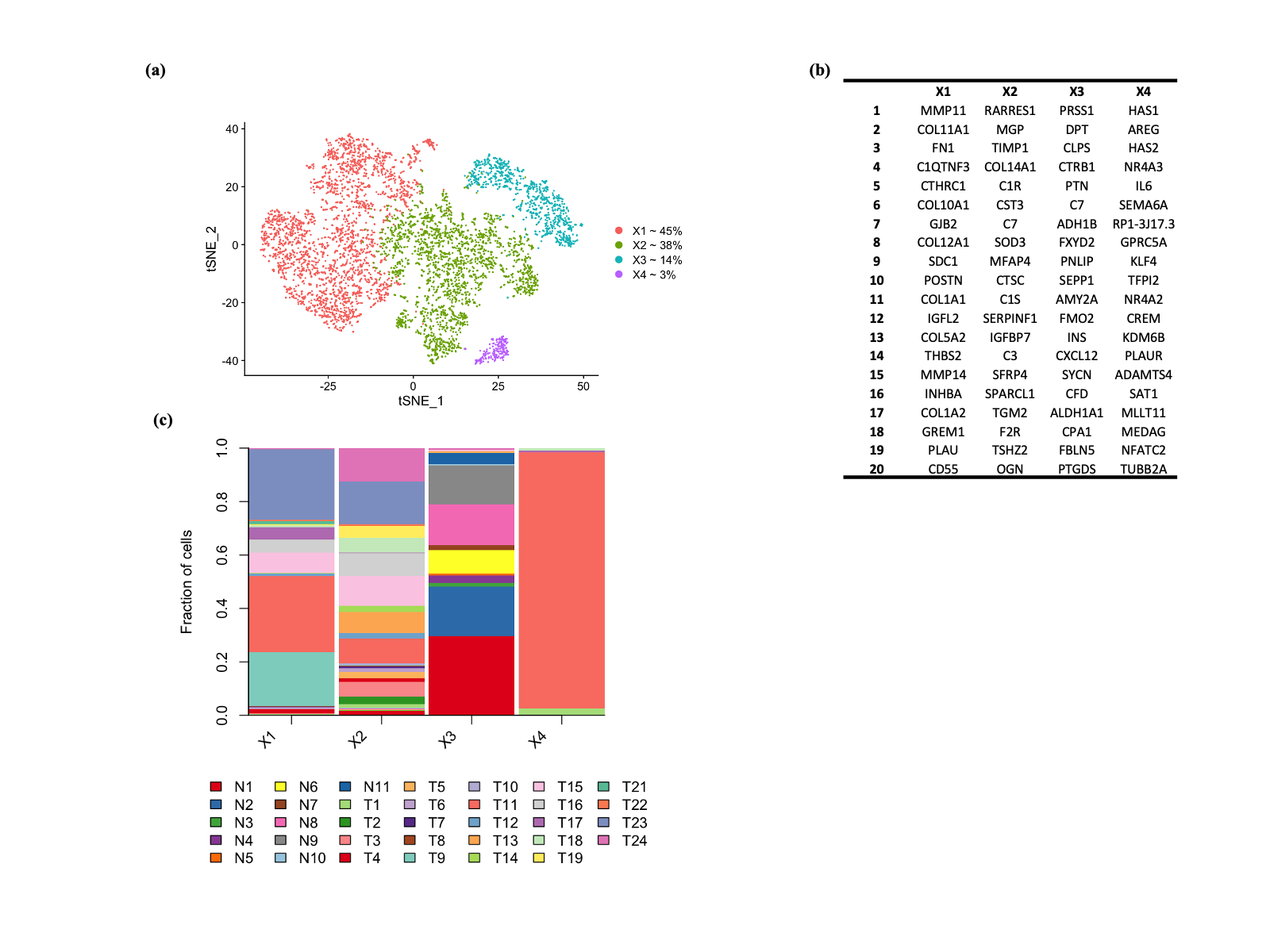

Supplement: S1 Fig — A. 6,267 fibroblasts originated from 11 control pancreases and 23 tumor samples were petitioned into four groups X1-X4. Fractions of the fibroblasts were: 45%, 38%, 14%, and 3%. B. Table showing the top 20 DE genes for each cluster. C. Bar plots presenting the numbers of cells captured for each cluster. (TIF) [file pcbi.1009228.s001.tif]

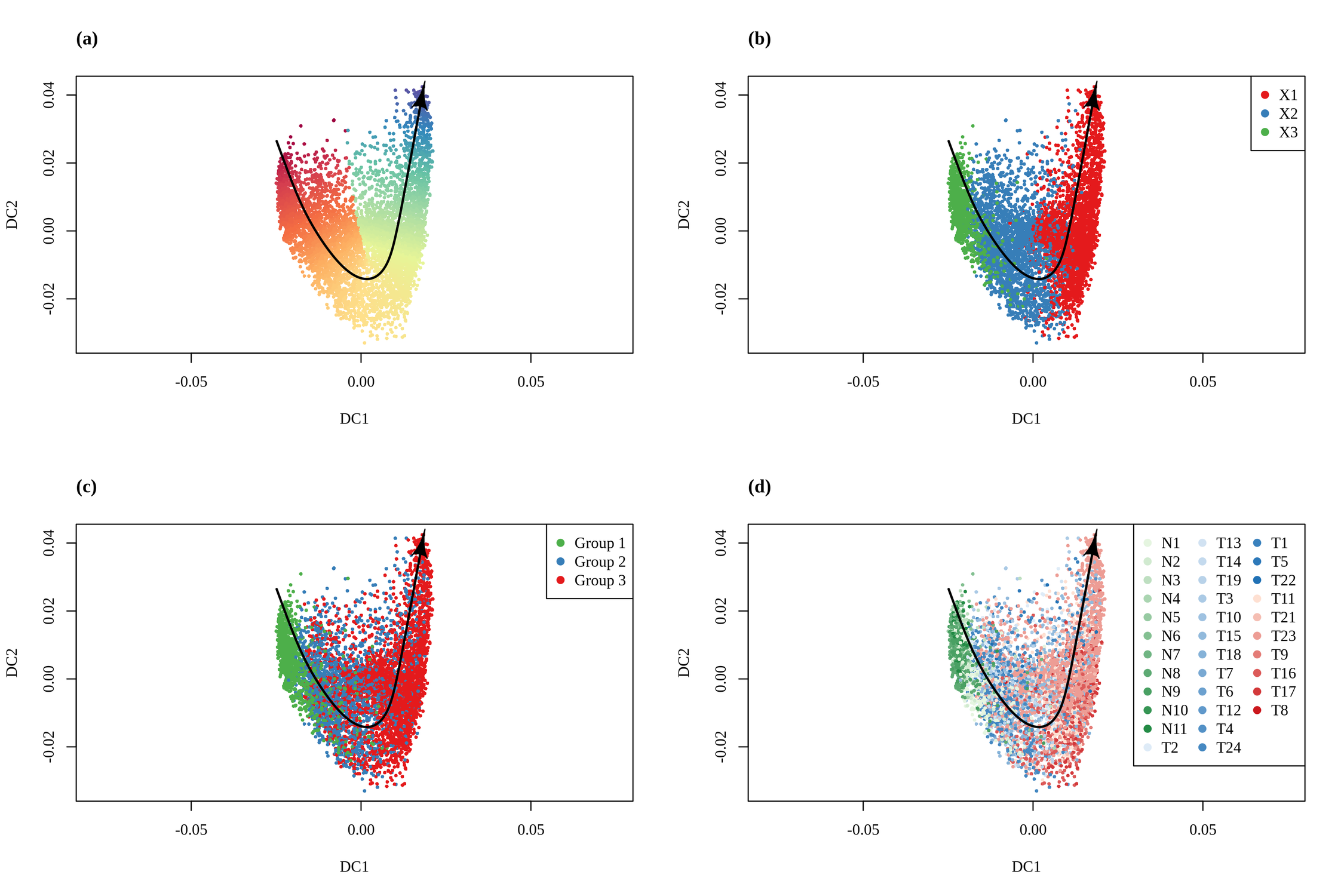

Supplement: S2 Fig — A. Colors coded for pseudotime changing, red presenting the beginning of differentiation and blue presenting the end. B. Color-coded trajectory analysis of fibroblasts for annotated three clusters. C. Color-coded trajectory analysis of fibroblasts for group information. D. Color-coded trajectory analysis of fibroblasts for sample identity. (TIF) [file pcbi.1009228.s002.tif]

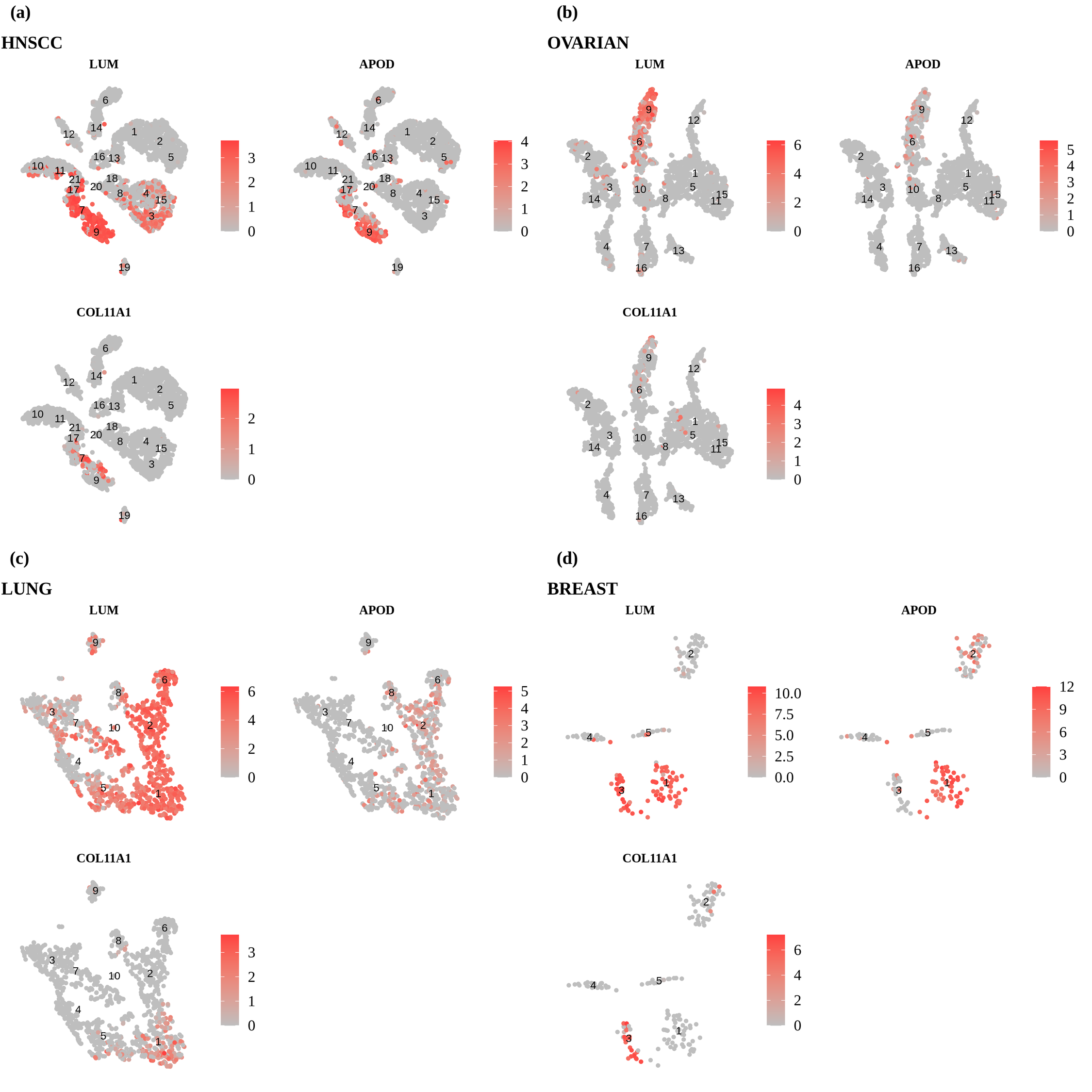

Supplement: S3 Fig — A. t-SNE embedding of the whole HNSCC dataset. B. t-SNE embedding of the whole ovarian cancer dataset. C. t-SNE embedding of the mesenchymal cells from lung cancer dataset. D. t-SNE embedding of the mesenchymal cells from breast cancer dataset. (TIF) [file pcbi.1009228.s003.tif]
